# A critique of the design, implementation, and delivery of a culturally-tailored self-management education intervention: a qualitative evaluation

**DOI:** 10.1186/s12913-015-0712-8

**Published:** 2015-02-07

**Authors:** Manbinder S Sidhu, Nicola K Gale, Paramjit Gill, Tom Marshall, Kate Jolly

**Affiliations:** Primary Care Clinical Sciences, University of Birmingham, Edgbaston, Birmingham B15 2TT UK; Health Services Management Centre, School of Social Policy, University of Birmingham, Edgbaston, Birmingham B15 2TT UK; Department of Public Health, Epidemiology and Biostatistics, University of Birmingham, Edgbaston, Birmingham B15 2TT UK

**Keywords:** Self-management, Education, Ethnicity, Lay, Culture

## Abstract

**Background:**

Self-management education is at the forefront of addressing the increasing prevalence of chronic diseases. For those at greatest risk, such as minority-ethnic and/or socio-economically deprived groups, self-management education can be culturally-tailored to encourage behavioural change. Yet, the application of culturally appropriate material and expertise within health promotion services continues to be debated. We critique the design, implementation, and delivery of a culturally-tailored self-management intervention, with particular focus on the experiences of lay educators.

**Methods:**

A mixed methods qualitative evaluation was undertaken to understand self-management service provision to culturally diverse communities (i.e. how components such as lay workers, group-based design, and culturally-appropriate educational material are intended to encourage behavioural change). We interviewed lay educators delivering the Chronic Disease Educator programme along with attendees, whilst observing workshops. Data were thematically analysed using a content-based constant comparison approach through a number of interpretative analytical stages.

**Results:**

Lay educators felt part of the local community, relating to attendees from different races and ethnicities. However, lay educators faced challenges when addressing health beliefs and changing lifestyle practices. Culturally-tailored components aided communication, with educator’s cultural awareness leading to close relationships with attendees, while the group-based design facilitated discussions of the emotional impact of illness.

**Conclusions:**

Lay educators bring with them a number of nuanced skills and knowledge when delivering self-management education. The development and training required for this role is inhibited by financial constraints at policy-level. The interpretation of being from the ‘community’ links with the identity and status of the lay role, overlapping notions of race, ethnicity, and language.

**Electronic supplementary material:**

The online version of this article (doi:10.1186/s12913-015-0712-8) contains supplementary material, which is available to authorized users.

## Background

Migrant populations are at greater risk of developing and living with long term conditions than the general population [1]. Further, migrant groups practice fewer self-management behaviours and demonstrate higher utilisation of emergency healthcare services in comparison to the general population [2]. One method to address the growing demand for health care services is by improving patients’ ability to self-manage by attending chronic disease self-management programmes (CDSMPs). Following a model developed by Lorig et al. [3] for patients living with arthritis, CDSMPs are weekly, group-based workshops delivered in primary care or community settings. Theoretically, CDSMPs intend to improve interaction with health care providers, making self-care decisions, managing the physical and emotional aspects of living with a chronic illness, and re-interpreting relationships with close others [4].

CDSMPs employ lay people (non-health professionals) from the ‘community’. In this context, lay people may be living with chronic diseases, have received self-management training, have knowledge of cultural beliefs and practices as well as societal issues facing the community. Within the UK Kennedy et al. [5], with their evaluation of the Expert Patients Programme (EPP), reported white, middle class, women are, particularly, attracted to volunteer lay positions as a method of accessing employment in the health care sector. Health policy has encouraged lay person recruitment according to ethnic concordance [6] and/or addressing language concerns. What remains inconclusive is how ‘being from the community’ enhances the experiences of those attending CDSMPs, and ultimately, encourage behaviour change in comparison to lay educators recruited elsewhere.

Cultural adaptation refers to the extent to which ethnic/cultural characteristics, experiences, values, behavioural patterns and beliefs of a target population as well as relevant historical, environmental and social factors are incorporated in the design, delivery, and evaluation of targeted health promotion materials and programs [7]. There remains a lack of evidence to guide practitioners on how best to culturally adapt interventions. Zeh et al. [8] have identified a number of cultural barriers for minority groups living with diabetes, where services need to address linguistic differences, different beliefs about health and illness, as well as addressing low concordance patients may have with western professional advice. Notably, Greenhalgh et al. [9] identified that among Sikh, Hindu and Muslim groups self-management was secondary to adhering to religious beliefs. However, some cultural barriers are more specific to certain ethnic groups and grouping all ethnic groups together, in terms of their health service needs, may be contentious [10].

Services are culturally tailored using five main strategies described by Kreuter and Wray [11]. These are: peripheral strategies (designing materials to appeal to a given group e.g. visual information); evidential strategies (presenting epidemiological evidence to raise awareness of health concerns), linguistic strategies (delivering in the dominant or native language of the target group), constituent-involving strategies (drawing on the experience of the group which includes hiring members of the indigenous population); and sociocultural strategies (discussing health-related issues in the context of broader social and/or cultural values).

These five main strategies for cultural tailoring fit within two broad categories with regard to adaptations that can be potentially used for interventions designed for minority-ethnic communities. First, cultural adaptation (adapting delivery or tailoring health information to reflect community values, beliefs, and practices); second, structural (modifying the intervention to encourage attendance and completion). The differentiation allows for the possibility to target specific components, where cultural adaptations relate to the nature of the content provided, and structural adaptations focus on issues of implementation. In addition, data collection with multi-ethnic populations can be problematic, as traditional ‘written’ methods are suited to the White population, whereas some South Asian sub-groups requiring assistance or audio delivery [12].

We evaluated a local service serving an ethnically diverse socio-economically disadvantaged inner city community. Our objectives were to i) describe the experiences of lay educators educating members of their own community with regard to self-management and ii) evaluate whether a culturally tailored self-management intervention is delivered as intended to an ethnically diverse population through observation and interpreting the views of completers.

## Methods

### Study design

To describe the experiences of lay educators and critique components of design, implementation, and delivery we undertook a theoretically-guided service evaluation. The objective was to evaluate whether components led to intended outcomes. For example, the group-based design is intended to enhance peer-to-peer exchanges amongst attendees, the application of visual aids and interactive tasks is expected to familiarise participants with low literacy, while lay educators are assumed to have greater knowledge of health beliefs and practices that can act as potential barriers to healthy living.

A qualitative approach was well suited to our evaluation, because qualitative methods can document and interpret the experiences of delivering and attending a self-management service. We collected both observational and interview data to gain multiple perspectives on the service. Semi structured interviews were conducted to understand lay educator’s experiences of delivering the intervention to an ethnically diverse population. We observed workshops delivered by each lay educator. Workshops varied considerably by: content, language in which content was delivered, and the characteristics of participants in a single group (ethnicity, gender, literacy needs). Therefore, it was imperative that as many different sessions were observed to interpret the various styles of delivery. Semi structured interviews were conducted with participants who had completed the intervention (attended at least 3 out of 4 workshops). Interviews were aimed at collecting data regarding participant experience and their views of lay educators.

### Setting

The intervention was delivered in Birmingham, UK. According to Quality and Outcomes Framework (QOF) data for April 2010- March 2011, for the local vicinity in which the intervention was delivered (74 practices, with 321 456 patients registered) the prevalence of CHD, diabetes mellitus, CKD were similar or greater than national levels.

### Intervention

The characteristics of lay educators/attendees interviewed and a description of the intervention are presented in Tables 1 and 2. We have used the Abraham and Michie [13] taxonomy to describe behaviour change techniques used within the intervention. The Chronic Disease Educator (CDE) programme was first delivered in March 2008 within general practitioner (GP) practices and community settings in Birmingham, UK. The intervention was delivered by a community interest company (CIC), providing lay health services to the local population. Patients suffering from diabetes mellitus (DM), coronary heart disease (CHD) and/or chronic kidney disease (CKD) were invited to attend.

**Table 1:** **Characteristics of participants interviewed/observed**

**Characteristic**	**Lay educators (N)**	**Attendees (N)**
**Gender**		
Male	2	11
Female	4	9
**Ethnicity**		
White British	1	7
White Other	-	1
Asian- Indian	-	5
Asian- Pakistani	2	1
Asian- Bangladeshi	1	2
Black Caribbean	1	4
Mixed Race- White British and Black Caribbean	1	-
**Age**		
Up to 29 years	4	-
30-39 years	2	-
40-49 years	-	4
50-59 years	-	3
60-69 years	-	10
70 or older	-	3
**Language**		
Bi-lingual	3	17
Non-Bi-lingual	3	3
**Years since diagnosis**		
Less than 5 years	-	7
5-10 years	-	4
10 years or more	-	9

**Table 2:** **Description of the chronic disease educator intervention**

**Reporting criteria**	**CDE Programme**
Where was the intervention delivered and why?	Primary care settings (GP surgeries) and community settings with a single lay educator (or with the use of interpreters when necessary
What behavioural change theory has intervention been based on (if any)?	Social learning theory
What behaviour change techniques were used by people delivering the intervention (if any)*?	1, 2, 4, 6, 8, 10, 19, 22, 24
A description of the activities and material provided in each workshop and their intended outcomes?	Material: information on weight management, choosing healthier foods, meal planning, physical exercise, checking and improving metabolic control and preventing complications.
	Activities: participants taking each other’s blood pressure, BMI calculations, understanding sugar and salt content in foods, Eat-well plate, food maps, guided imagery, ‘freethink’.
	Intended outcomes: desirable body weight, learn to shop for food, increase physical activity, take medication properly and regularly, recognise early symptoms of condition, regularly attend clinics, improved symptom control, reduced BMI, improved quality of life and knowledge of condition, reduced level of prescribing, slower disease progression, management of condition, carry out normal roles and activities, and manage emotional impact of illness.
What support (if any) was provided to individuals outside of workshops?	No contact outside of workshops.
Was a manual or protocol used to deliver the intervention and are there details on how it can be accessed?	Manual is available to lay educators, however, not used during intervention. Can be accessed via permission from Health Exchange.
How were individuals referred to the programme?	Patients suffering from diabetes mellitus, coronary heart disease and/or chronic kidney disease were invited to attend the programme. Patients referred to the programme by general practitioners, practice nurses or practice staff. Practices citing a lack of time asked CDEs to contact patients on chronic disease registers directly by phone or postal mail.
Were any cultural or structural adaptations used?	Delivery in various languages, cultural adaptation of educational material, application of visual aids and demonstrations, understandable terminology, emotional well-being, culturally sensitive approach to delivery, recruitment of lay personnel, delivery in community locations, and religious/cultural acknowledgement.

*Abraham and Michie [13] Taxonomy of behaviour change techniques:

1. Provide general information on behaviour-health link; 2. Provide information on consequences; 3. Provide information about others’ approval; 4.Prompt intention formation; 5. Prompt barrier identification; 6. Provide general encouragement; 7. Set graded tasks; 8. Provide instruction; 9.Model/Demonstrate the behaviour; 10. Prompt specific goal setting; 11. Prompt review of behavioural goals; 12. Prompt self-monitoring of behaviour; 13. Provide feedback on performance; 14. Provide contingent rewards; 15. Teach to use prompts/cues; 16. Agree behavioural contract; 17. Prompt practice; 18. Use of follow-up prompts; 19. Provide opportunities for social comparison; 20. Plan social support/social change; 21.Prompt identification as role model/position advocate; 22. Prompt self-talk; 23. Relapse prevention; 24. Stress management; 25. Motivational interviewing; 26. Time management.

The intervention was culturally-tailored and differed from the CDSMP model developed by Lorig et al. [14]. The service was delivered over four weeks, once a week for 90 minutes each time, by a single lay educator. Participants were allocated to groups (3–15 people) according to language requirements, with non-English-speaking participants (exclusively South Asian individuals) allocated to groups with a bi-lingual lay educator (Punjabi, Urdu, Hindi or Sylheti) or if a bi-lingual educator was unavailable, an interpreter with necessary language skills was used. Participants were referred to the programme by GPs, practice nurses, practice staff or lay educators.

Content included information on weight management, choosing healthier foods, meal planning, physical activity, checking and improving metabolic control and preventing complications associated with chronic diseases. The programme content was underpinned by social learning theory [15] and included skills mastery, action planning, social support (via the group), goal setting, and problem solving. To accommodate participants from socio-economically disadvantaged backgrounds with low literacy levels the programme used visual aids and demonstrations, understandable terminology, and was delivered in community locations.

### Fidelity of the intervention

Lay educators were provided with a ‘manual’ detailing the aims, content, and goals that should be completed in each workshop of the intervention. For example, lay educators were expected to inform attendees of the aim at the beginning of each workshop. Lay educators were assessed on: whether there was group interaction between participants and educators, use of appropriate cultural adaptations for the demographic characteristics of the participants, appropriate translation, supporting self-efficacy, setting goals and reviewing progress against targets.

### Recruiting lay people from the community

Lay educators were recruited from the local community. These were people from different ethnic communities, who lived in Birmingham and had knowledge of local social issues. At the beginning of data collection six lay educators were delivering the programme. Training was provided in partnership with a local college, with lay educators completing a national vocational qualification in health and social care.

### Sample

All six lay educators agreed to be interviewed. A purposive sampling method was used with regards to observing the CDE programme and generating a sample of participants that completed the intervention [16]. All sessions (workshops 1–4) were observed for a single bi-lingual and mono-lingual educator, while the first and last workshops were observed for the remaining educators. We observed workshops delivered in various community languages, to men and women, and to different ethnic groups to understand the acceptability of the intervention for different sub-groups. Completers of the programme invited for interviews were purposively sampled by ethnicity. In order to ascertain whether the intervention was delivered as intended we wished to gather the views of participants completing the intervention, to identify what they felt was positive or could be improved about the intervention, with regard to content and delivery, for future service users. Lay educators asked participants attending workshops whether they would like to be interviewed and share their views about the intervention.

### Data collection

MS completed semi-structured interviews with lay educators (N = 6) and a sample of participants who completed the CDE programme (N = 20). MS is a sociologist by background (BA Hons at the time of data collection). Interviews with lay educators were conducted at their place of work, while interviews with participants took place in their homes or a meeting room at the Central Library, Birmingham. All interviews were completed by MS. An interview guide was used (different for lay educators and participants, Additional files 1 and 2), based on a review of the literature and discussions within the research team (MS, NG and KJ). With reference to the work of Brown et al. [17] and Tozer et al. [18], the interview guide was structured about the lay educator role, its responsibilities, training and the experience of being and working within the local community. The interview guide for participants was thematically designed incorporating questions about style of facilitation, meeting cultural/language needs, application of behavioural change techniques, and delivery of content. A key focus was placed on understanding the interactions amongst participants during group based education and whether this method was acceptable for attendees. Data collection ceased when no new descriptive themes were emerging from interviews. All interviews were audio-taped and transcribed verbatim. Three interviews with participants were in Punjabi or Urdu and one conducted with an interpreter and transcribed in English (Bengali speaker). Translation was independently checked for conceptual equivalence by another researcher with appropriate language skills.

To develop an understanding of the CDE programme we observed educational workshops (N = 14 [workshops]). MS observed all sessions. There were three areas evaluated for fidelity: observing the nature of interactions between participants with each other and educators, the approach and language (literally and conceptually) used by educators, and whether cultural and social norms (in relation to healthy living) were addressed. The role of observer as participant (the researcher has minimal interaction in the research setting) was chosen [19]. Observational data were recorded using a thematically-designed research instrument categorised into programme delivery and participant interaction. The instrument (Additional file 3), designed by the research team, was developed to collect data with regard to group dynamics, behavioural change techniques, variation in language used, delivery styles and cultural competency of lay educators.

### Data analysis

Data analysis occurred in tandem with data collection. MS conducted and transcribed all interviews (English and non-English) and recorded data from observations. Transcriptions of non-English interviews were verified for conceptual equivalence with another researcher within the university with experience of conducting interviews in South Asian languages. With regard to reliability, a purposive sample of transcripts was independently coded by NG (medical sociologist). The intention was to independently identify areas of interest within data that could lead to the generation of themes. Monthly (NG and KJ) and bi-annual meetings (PG and TM) to discuss the generation of themes were held to increase methodological rigour [20]. Using a constant comparison approach, we used applied thematic analysis via an inductive process. We used content analysis and themes emerged from the data inductively. In an attempt to be systematic throughout, our coding was completed in a number of cycles, iteratively moving back and forth between data collection and analysis.

First cycle coding involved reading transcripts [and observational data], identifying data of interest and encoding prior to interpretation [21]; hence, codes were created that “summarize, synthesise, and sort many observations made of the data” ([22]: 112). Once complete, the second cycle involved the application of axial coding; placing relating codes together that are based on a single phenomenon and making connections. Throughout axial coding, we adopted a technique of writing brief analytical memos to detail the development of categories, make relationships between codes, facilitating theoretical interpretation, and linking findings with the literature. Finally, themes were generated by writing initial descriptive themes, then clustering themes together to generate an inductive deduced integrative theme. NVivo software was used to assist data analysis.

### Consent and ethical issues

Ethical approval was obtained from South Birmingham Research Ethics Committee. Participants were given written information about the evaluation and gave written informed consent. Quotes have been given identifiers to ensure anonymity.

## Results

### Findings: Lay educators

A table of themes and supporting quotes are attached at the end of the manuscript (Additional file 4).

#### Cultural receptivity

Lay educators interpreted themselves as knowledgeable experts, where knowledge and information they disseminated was perceived to be of greater value compared to health beliefs held by participants on the programme. Participants were allowed to discuss their health beliefs; however, discussions were concluded by distinguishing whether a belief was ‘fact’ (conformed to scientific-based evidence) or ‘myth’ (related to folk beliefs). This was complemented by participants placing greater value on information given by lay educators, particularly about healthy foods, in comparison to other members of the group:

They just ask me, “is this true, is this true”, I go this is what’s true and some are myths, so try not to follow the myths just the facts (CDE, interview).

Their [White British and Black/Black Caribbean] mentality is slightly different to the Asian culture so you know they don’t eat many spices anyway, their lifestyles, their little habits. I find that’s its easier for them to make the changes than it is of Asian people because they just have their set plans you know, you make one dish and you have it twice a day, and everybody has the same and you have your chapattis (CDE, interview).

Cultural adaptations were made, in their majority, to meet the needs of South Asian participants attending the programme. Activities were adapted by incorporating established Asian brands when discussing cooking practices and nutritional value. Female only groups were delivered for Muslim participants on request:

We show them the traffic light system, like types of food, so instead of using English brands I use Asian brands, like East End (CDE, interview).

if it was Muslim women, sometimes they prefer a women only group so you have to be sensitive to that (CDE, interview).

The activities on the CDE programme were culturally adapted to include visual aids for illiterate and/or poorly educated participants to get across the message of healthy eating. Visual aids were not always applicable to certain ethnic groups, where they would be more useful if they were culturally orientated to meet particular needs for individual communities:

the visual aids aren’t culturally relevant, like with the food plate the oils and fats that we’ve got, even the bits of salt aren’t culturally relevant… the oils we purchased from an outside agency, the salt’s again have been purchased by an outside agency as well, so it’s what they provide (CDE, interview).

#### Working with and without interpreters

The need to establish a productive working relationship between lay educator and interpreter was imperative, particularly to ascertain that their roles did not overlap. Mono-lingual educators recognised the importance interpreters played when there was no ethnic concordance with participants. As interpreters were ethnically matched with participants, they acted as a link and helped to break any perceived barriers of us and them:

The interpreter that I work [with], they (South Asian community) really value her, they do, I suppose they’re also seen as part of her community as well. Us working alongside together has been, very positive. I suppose it has broken down perhaps a barrier that may have been between me and the group in a way, if that was ever felt (CDE, interview).

For South Asian bi-lingual educators (N = 3), speaking a second language provided the opportunity to apply contextual knowledge about beliefs, practices and experiences of living with chronic diseases. However, being ethnically, rather than linguistically matched, with members of the South Asian community resulted in a number of difficulties. There were issues with multiple community languages being spoken in a single workshop:

I have to be very strict and say I’m going to do a language only course, cause it’s very hard to chop and change, we tried it and it doesn’t work…cause it would be a lot easier delivering it in a community language than it is delivering half in a community language then in English and back into a community language, cause people just get frustrated, they get tired (CDE, interview).

Lay educators were faced with the experience of dealing with practices that were culturally ingrained while simultaneously delivering health information that was medically validated. Therefore, there were difficulties trying to translate health education messages designed for western lifestyles for South Asian communities:

It’s quite tiring because working with Asian people is a challenge, because I find it very challenging, because it’s a very tough community to work with and especially trying to send out the message that we want, because they’re so set in their ways (CDE, interview).

Being ‘from the community’ addresses surface level cultural needs such as establishing clear lines of communication through a single language. Conversely, validated health education messages based on western diets and lifestyles were difficult to translate and considered culturally irrelevant which led educators to adapt content to fit participant lifestyles:

they’ve all got their individual problems, for example Jamaican’s they tend to use a lot of salt on their salt fish and everything, you’ve got Asian’s with the fat and the *ghee* [clarified butter] (CDE, interview).

### Findings: observations for fidelity

#### Use of appropriate cultural adaptations

Visual aids were perceived to be valuable when educating South Asian members of the group. The CDEs felt that, in general, South Asian people in their groups had lower baseline knowledge of the content provided on the programme compared to people from White British and Black Caribbean communities. For example, CDEs would first explain what the different food groups are before activities using a food mat or food traffic light system were carried out with South Asian participants. The use of visual aids was able to make the ‘take home message’ of the activities much clearer and easy to understand for members of this group:

The use of the visual aids (images of food portions and the sugar bags- identifying how much sugar is in different types of food) makes a considerable impact on providing a complete picture and makes the content very relevant. PT (1) responds in English, “My God”- PT (5) responds in Punjabi and English combined when she sees a picture of some ice cream, “*bhoort tasty*” [*very tasty*]- CDE picks up image and replies that it is very sugary and unhealthy- PT (1) takes sugar bag- PT (3) in Urdu, “*herani*” [shocked/amazed] (CDE, observational notes).

#### Setting goals and reviewing progress against targets

Goal setting was primarily performed at the end of the intervention (week 4) rather than setting goals in each session based on the content covered. Furthermore, goal setting was vague and non-personalised; for example, encouraging participants to lose weight but not distinguishing how much weight to lose and over what length of time. Food diaries were inappropriately used on the programme. Rather than identifying potential areas for change in a participant’s diet they were seen as a tool that could be used by individuals in isolation. Therefore, food diaries highlight a potential area where personalised behavioural changes can be made with the help of the CDE:

No recap of the previous week- all participants present in this session were present in the previous session- PT 3,6,8,9,13 bring their food dairies to the session- CDE does have a look at these diaries, however, gives the diaries back to them, “it’s for you guys to use really”. Some appear to think that it was bit of a useless task, maybe wished to gain more feedback from the CDE; advice on where changes could be made, more knowledge and guidance (CDE, observational notes).

CDE goes round the table- not asking everyone- on what changes they have made since they began the course- PT 3- “look at labels, more oats, trying to cut down the salt” PT 2-“I’ve learnt a lot, cut down on the chocolate, started telling everyone else” (CDE, observational notes).

#### Group interaction between participants and educators

Throughout workshops there was a shift between the roles of an ‘educator’ (providing health-related information through a didactic approach) or a ‘facilitator’ (encouraging participants to direct sessions in areas they felt were relevant and narrating personal experiences). Educators were comfortable in an ‘expert-led’ position which allowed them to maintain greater control in sessions. This position allowed parameters to be set around group discussions i.e. ensuring participants discussed the content covered which could be personalised:

Discussion about tension [stress], thoughts and sadness. CDE informing PT 1 to take care of his blood pressure- caring daughter approach (CDE, observational notes).

Participants were able to interact with each other at ease, once lay educators had set the topic of discussion. Small groups formed, primarily by gender, where both men and woman felt more comfortable discussing lifestyle behaviours that they felt were gender specific, for example weight maintenance. Yet, interactions were directed towards being more informed so instigating lifestyle changes:

PTs are very interactive with those who are closest to them (PT 13, 7, 6) (PT 8 and 9) and (PT 2 and 3): small groups have formed. PT 6,7 very interactive- PT 5 not present- PT 13 joins this group- females of her age- feels she can relate to them better. PT 13 asks PT 7 if she would like to go to the gym with her- has no one to go with- PT 7 declines- problem with self-image- in front of others (CDE, observational notes).

PT 2 and 3 (both male)- in conversation for a long time- conversations lead to questions directed to the CDE- all related to the discussion- salt causing high BP- giving each other more information (CDE, observational notes).

#### Supporting self-efficacy

Discussions with participants were occasions when behavioural change techniques, underpinned by social learning theory, were applied. The most common technique was encouraging self-efficacy i.e. improving an individual’s perception that they can successfully perform a behaviour that will have successful outcomes:

CDE stated that going out, socialising, and going to church encourages positive well-being. Participants already confident of undertaking tasks that will improve their emotional well-being (CDE, observational notes).

The use of persuasion. Outlining the benefits of undertaking certain tasks such as exercise which can improve stress management and (CDE) stating to the participant more exercise will result in a positive outlook; participant nods in agreement (CDE, observational notes).

#### Appropriate translation

Workshops delivered in Urdu and Punjabi, either through an interpreter or a bi-lingual educator had a ‘stop-start’ feature. Workshops with an interpreter often involved the educator speaking in English and then the interpreter translating information into Punjabi and, at times, adding more information than what was actually said. The interpreter would often answer patient queries directly as a result of previously working on the programme. CDEs would struggle to control interactions as participants directed their questions to the interpreter rather than the CDE. This often resulted in CDEs explicitly asking what was being said in discussions:

Sorry, so what’s being said? (CDE, observational notes).

The use of interpreters and participants with various language requirements created a ‘chain of translation’, where the information is first delivered by the educator in English, translated into Punjabi by the interpreter and then further translated into another community language e.g. Bengali, by participants to other members of the group, with information being lost along the chain:

PT 5 states she did not understand the translation delivered to her by the interpreter- firstly in Punjabi, and then does it in Urdu- slightly better. PT 5 speaks Bengali- not spoken by the interpreter which did make her slightly isolated (CDE, observational notes).

Workshops delivered in a single language tended to have less of a ‘stop-start’ feature. This allowed discussions to develop from activities which led to greater participation from the majority of people in a group.

### Findings: the participant experience

The promotion and development of chronic disease self-management programmes has led to an array of terms being used to describe lay people, such as peer mentors, peer leaders, peer educators, lay health workers, community health workers, community health educators and many more. Unsurprisingly, participants were unfamiliar with the term ‘Chronic Disease Educator’ or the role:

The impression I got with him … I was a bit apprehensive about him, but when he started talking … I think if you need to ask him, he was there to ask (CDE-ATT-17, Male, White British, T2D and heart conditions).

Lay educators and participants developed relationships that were trusting, consisted of rapport and empathy. In part, the relationships established were, paradoxically, given value and meaning through comparison with the relationship participants had with their GP. Participants were asked whether lay educators recruited from the community influenced the nature of the relationship which ensued. Ethnic concordance, for some was valued less than other characteristics such as being understanding:

[Interviewer] So, was it of any benefit that [CDE] was from a similar background?

[Respondent] No, whoever’s a good person, like we speak to white people, we speak to Black people, so what’s wrong if everybody is friendly with each other (CDE-ATT-13, Female, Asian Indian, heart conditions).

South Asian respondents, overall, valued lay educators from their community because they were able to deliver the course in community languages. Instances where there were ethnic differences (Indian Sikh participant/Pakistani Muslim educator) made little or no difference, with the exception of one person who felt a ‘bit more comfortable that she [CDE] was Pakistani’ (CDE-ATT-12, Female, Asian Pakistani, T2D):

She was telling everyone in Punjabi, do this, do that, she spoke good Punjabi, sometimes she spoke in English, everything was fine with her (CDE-ATT-11, Female, Asian Indian, T2D and Hypertension).

For the majority of participants, their relationship with lay educators was determined by their prior knowledge of self-management. Primarily, there were two types of relationships; first, informal and ‘friendly’, and second, a didactic teacher-pupil relationship with a clear intention of learning:

He was mostly happy with the fact that on how she [CDE] advised on how to lose the weight, how to minimise your food [consumption] and stuff because he wasn’t very aware of it (CDE-ATT-07, Male, Asian Bangladeshi, T2D, via interpreter).

No, no it didn’t feel like a classroom at all, no, it was just like you were going in to have a talk with a normal friend in a friendly atmosphere (CDE-ATT-03, Female, White British, T2D).

Members from various ethnic groups described experiencing different types of relationships. White British and Black/Black Caribbean interviewees described the relationship as informal, based on personal conversations, friendliness, and listening. In contrast, South Asian interviewees described a formal relationship based on learning and developing skills that would potentially improve the management of their conditions. South Asian participants appeared content with a ‘teacher-pupil’ relationship acknowledging that the lay educator was better informed and should lead workshops.

Peer-to-peer exchanges were limited to sharing personal narratives, discussing past experiences of living with and managing conditions, and relieving emotional distress. Participants listened with scepticism to the narratives of other group members. For some, self-management of chronic diseases, particularly diabetes, was interpreted as a personalised experience, for example, participants living with diabetes spoke of dealing with hypoglycaemic episodes:

if you ask another person with diabetes, he will tell you his experience, you could ask a hundred people, if you talk to a hundred people they all give you their advice, of their experience, but sometimes it doesn’t work with you, you know (CDE-ATT-15, Male, Asian Indian, T2D).

Because we’re all individuals, what suits me, they could not copy me because it may not suit them (CDE-ATT-04, Female, Black Caribbean, T2D).

The whole condition was brought out in the group because people were at different stages with their diabetes and on different medication and they were experiencing different things because a lot of them, or some of them, had different health issues, medical issues, as well as their diabetes (CDE-ATT-10, Female, Black Carribbean, T2D).

Notably, when participants shared a personal narrative there was a lack of facilitation from lay educators. None of the lay educators had the experience of living with chronic diseases. Greater facilitation by concentrating on positive experiences e.g. appropriately dealing with hypoglycaemic events, rather than negative experiences could potentially result in greater peer-to-peer learning.

## Discussion

### Design

The group-based format of the programme allowed participants to share personal experiences of living with chronic diseases but were unstructured and without a specific goal in mind. In comparison, Greenhalgh et al. [23] ‘sharing stories’ intervention had a clear focus on a topic of discussion (medication), participants sharing problems (specific issues with taking a combination of drugs), identification of common problem (group members not taking medication), and an outcome (telling GPs about non-concordance and GP informing participants how to deal with side effects from drugs). The group-based approach lacked an exchange of skills and/or learning from each other’s experiences.

Analysis of participant accounts has shown that people from certain ethnic communities have different learning requirements and skills they wish to develop. A review by White *et al.* [24] investigating the effectiveness of interventions to promote healthy eating in people from minority-ethnic groups found behavioural modification was more effective in European origin (White) groups compared to minority-ethnic groups. Interventions accommodating South Asian groups should concentrate on addressing cultural barriers to effective self-management, specifically addressing commitment to religious beliefs, linguistic differences between patients and health workers, and low literacy levels [8].

### Implementation

Understanding the role of lay educators was important for participants, as it influenced the type of role they adopted within workshops and their interpretation of the CDE programme. The relationship between participants and CDEs was contextualised and understood in relation to perceptions towards the quality of care they expected to receive from health professionals. As a result, the relationship was performing a core aspect of their perceived ‘doctor-patient’ relationship; an on-going personalised therapeutic relationship based on listening and allowing the patient to have a greater say in their treatment [25]. Lay educators (and organisations delivering CDSMPs) need to demonstrate how this programme fits within wider health services. Lay educators may benefit from formally being introduced in a team model with health professionals (GPs, practice nurses); with lay people, from the local community, playing a valuable role by providing contextual information about a patient’s attitudes, behaviour, and environment [26].

### Delivery

The training needs of lay people delivering group-based health education to minority-ethnic disadvantaged populations need to be examined. There were a number of areas where training could be provided: how to communicate with health professionals, facilitating discussions with adult learners, and how to work with interpreters. In contrast to findings presented by Brown *et al.* [17], lay educators did not express any need to develop their knowledge on healthy living or chronic diseases as they were comfortable dealing with health beliefs held by members from various ethnic communities. Hipwell et al. [27] noted the need for cultural competence training i.e. enhancing tutor confidence when dealing with multi-ethnic groups. Through observation of the CDE programme it was clear lay people delivering self-management interventions need to be adequately trained (or undertake further training) in techniques which encourage a person to change their behaviour. The techniques that need to be developed are:

1. recognising which behaviours to change,
2. making explicit the health benefits of making changes,
3. setting realistic targets (use of SMART goals or action planning),
4. reviewing targets, and
5. informing individuals how to deal with potential setbacks.

The definition and application of these techniques have been explained in *Improving Health: Changing Behaviour, NHS Health Trainer Handbook* [28]. Recruiting lay educators from the community benefited members from the South Asian population most compared to other ethnic groups. South Asian participants who attended the CDE programme benefited from improved communication while Black Caribbean and White participants benefited from establishing close understanding relationships. Ethnic concordance is thought to reduce the potential for power disparities between patient and health care provider, consequently patients becoming more involved in the decision making process. Yet, in this programme ethnic concordance had little or no difference with regard to participant involvement, particularly with South Asian participants.

### Strengths and limitations

Strengths of our study are the use of mixed methods, the application of research instruments that were thematically designed with reference to self-management literature and data were collected from a diverse sample of different ethnic and religious groups, male and female and living with a range of chronic diseases. Participants with language requirements were included within the sample (Punjabi, Bengali and Urdu speakers). There were a number of limitations. Participants, in their majority, came from courses delivered by three lay educators. For some interviewees there was also a considerable amount of time between completing the course and conducting the interview which inevitably affected what participants could remember about attending the programme.

## Conclusion

Understanding the role of educators delivering self-management interventions within the UK is under-developed, particularly with minority-ethnic populations. Analysis of our findings show greater considerations need to be made in relation to designing content, methods of implementation, and on-going training for lay educators when facilitating behavioural change. Lay educators recruited from the community bring a number of skills, particularly knowledge of health beliefs and practices, yet, for participants attending self-management interventions, skills such as empathy, understanding, and providing health information bypassed issues of ethnic concordance.

## Additional files

Additional file 1:
**Research Instrument for Chronic Disease Educator Semi-Structured interviews.**
Additional file 2:
**Research Instrument for Chronic Disease Educator programme: Participant interviews.**
Additional file 3:
**Research instrument for observation.**
Additional file 4:
**Themes and supporting quotes.**
