# Supplementary material for: A critique of the design, implementation, and delivery of a culturally-tailored self-management education intervention: a qualitative evaluation
Source: BMC Health Serv Res. 2015 Feb 7;15:54. doi: 10.1186/s12913-015-0712-8 (PMC4326406; doi:10.1186/s12913-015-0712-8)
Supplement: Additional file 1: — Research Instrument for Chronic Disease Educator Semi-Structured interviews. [file 12913_2015_712_MOESM1_ESM.doc]

**Additional file 1: Research Instrument for Chronic Disease Educator Semi-Structured interviews**

Participant Details

Participants name:

Age:

Location of session:

Ethnic origin:

Whether they live locally:

Date they began employment with Health Exchange:

Completed Foundation Degree at Matthew Boulton College (MBC):

Background

1. Can you tell me how you came to know about Health Exchange and the role you are currently doing?
2. Have you previously worked in the health sector before this position? *(ask only if this question has not been answered in the previous question)*
3. Why did you decide to become a Chronic Disease Educator? What was attractive about the position?

Structure of the Sessions/Programme

1. How are the CDE sessions planned- do you tend to follow the Health Exchange handbook?
2. Do you make any adjustments to the session content/layout? *What are they- cultural, social, religious changes? Does the CDE have any influence on the content of the sessions- would they like to have greater involvement?*
3. Do you have to meet any targets within the programme? *(what are these targets, how does it affect their role- pressure, less time educating)*

Group Dynamics

1. How do you like to deliver your sessions to the patients involved in the programme? *(one-to-one, group activities, lecturing, mixing pts into groups, making it enjoyable- informal approach- jokes)*
2. Do you actively seek to mix groups? *(why- more effective in changing pts behaviour and/or attitudes- how- ethnicity, language, age, gender)*
3. Are you wary of one person dominating the group- can you give an example?
4. What did you do to tackle any possible power relations?

Language

1. Are there any advantages or disadvantages for being bi-lingual (or non-bi-lingual)?
2. Is the session more or less effective through the use of interpreters?
3. Are the interpreters simply seen as an aid to the sessions?
4. Which groups or patients do you feel that you have the biggest impact upon when trying to change their dietary behaviour and attitudes? *(is language or ethnicity a fundamental factor in this?)*

Attitudes

1. What are your opinions upon the content of the sessions? *(easy/hard to teach, will it have an impact on patients, too ‘wishy-washy’)*
2. How do you feel about teaching these sessions to the community that you are from? *(more comfortable/ uncomfortable)*
3. Would you feel more comfortable teaching to another group of patients? Why?
4. What are your opinions of the Health Exchange Management? *(initially, have they changed, are they helpful-how- what would they change)*
5. Overall what are opinions of the programme itself? *(is it simply a stepping ladder for something else or long term position?)*

Theoretical Application

1. Are there any barriers to understanding for the patients?
2. What do you think the biggest barriers to understanding are for the patients involved in the programme? *(does not have to be programme specific- incorporate other social factors if possible)*
