# Supplementary material for: A critique of the design, implementation, and delivery of a culturally-tailored self-management education intervention: a qualitative evaluation
Source: BMC Health Serv Res. 2015 Feb 7;15:54. doi: 10.1186/s12913-015-0712-8 (PMC4326406; doi:10.1186/s12913-015-0712-8)
Supplement: Additional file 2: — Research Instrument for Chronic Disease Educator programme: Participant interviews. [file 12913_2015_712_MOESM2_ESM.doc]

**Additional file 2: Research Instrument for Chronic Disease Educator programme: Participant interviews**

Participant Details

Participants name:

Age:

Ethnic origin:

Background on patient

1. I understand you were invited to the CDE programme which is run for people with heart disease, diabetes or kidney disease. Can you tell me about the condition that you have?

*How long have you suffered from this?*

*(Sub-questions)*

*What are your conditions?*

*What were you doing to manage your condition?*

*What impact was it having on your life- social; work; family*

*Had you used any other NHS services?*

Reasons for participating in a group-based health education programme

1. What were your expectations of the programme? (immediate relief; cure; greater knowledge; tips and techniques

*(Sub-questions)*

*Advice on specific problem with condition*

*Wished to help others*

Group-based education

1. Can you tell me what happened within sessions? (the role of the educator; the patients role; activities; leaflets)
2. How did you feel about the group approach? (talking in front of men/women; members of the same ethnic group; prefer 1-2-1 style)
3. Could you relate to other members of the group? (*female/male; similar condition; ethnicity/ religion; age- peer identification)*

Content and delivery

1. What were your opinions of the educator? *(the style in which they delivered the information; were they empathetic; were you able to build a bond with the educator)*
2. What did you make of the way the information was presented? *(didactic; participative; encouraging; humorous; teacher-like)*
3. What aspects of the programme did you most enjoy? *(learning new information; improve emotional well being; meeting new people suffering with similar conditions as themselves; being given the opportunity to narrate their personal experiences)*
4. What aspects of the programme did you least enjoy? *(dominant group members- not having the opportunity to talk; filling in questionnaires; group size- too large/ too small)*

Application of health information

1. Did you learn anything new from the programme? (what was it, has it made an improvement in their condition)
2. Who did you learn this information from? (educator; peers- the level of trust- assured)
3. How do you feel about managing your condition since the programme? *(positive outlook; no change; empowered; greater control; have their been any improvements- GP, social, psychological)*
4. Would you recommend this programme to other people? *(why/why not- positives/negatives- to which type of person would they recommend it to)*
5. What further support would you like now? (personalised approach; individual/group; seeing the same group again; more medical information; was this programme enough)
