# Supplementary material for: A critique of the design, implementation, and delivery of a culturally-tailored self-management education intervention: a qualitative evaluation
Source: BMC Health Serv Res. 2015 Feb 7;15:54. doi: 10.1186/s12913-015-0712-8 (PMC4326406; doi:10.1186/s12913-015-0712-8)
Supplement: Additional file 3: — Research instrument for observation. [file 12913_2015_712_MOESM3_ESM.doc]

**Additional file** 3: Research instrument for observation

| *Variable under Consideration* | *Area of Investigation* | *Time of observation* | *Observation* | *Further Questions/ Areas to investigate* |
| --- | --- | --- | --- | --- |
| Background Information.  (1) | Location:  Date and Time:  CDE: e.g. South Asian, male  CDE Session: e.g. Introduction (1) |  |  |  |
|  | Interruptions: |  |  |  |
| Group Dynamics  (2) | Number of Participants: |  |  |  |
| (3) | Characteristics of the group? |  |  |  |
| (4) | Does the CDE mix individuals up?  *(ethnicity, gender, age, language- forced, reluctance, volunteer)* |  |  |  |
| (5) | Are there any groups that have formed? *(by the CDE, naturally)* |  |  |  |
| (6) | Does the CDE bring in individuals who seen to be ‘semi- excluded’? *(who is excluded, why, what is done, one-to-one approach)* |  |  |  |
| (7) | Is there any patient ‘mapping’ to those that similar to them? *(similar patients interacting together)*  *Peer identification* |  |  |  |
| (8) | Are patient questions answered through the group or the CDE?  *(evidence of patients informing each other, information through the CDE only)* |  |  |  |
| Use of Language  (9) | Is the CDE bi-lingual or English speaker only? |  |  |  |
| (10) | Is there an interpreter present? |  |  |  |
| (11) | Is the session constructed on the basis of language? *(separate those that need interpreter, slower pace)* |  |  |  |
| (12) | Does the CDE adopt a formal/informal approach in regards to his/her language? |  |  |  |
| (13) | Does the mixture of words provide a more complete picture? *(is it a useful aid in teaching/ hindrance to others)* |  |  |  |
| (14) | Does the use of multiple langs break/create barriers to entry? *(confidence/confusion/inclusion/*  *exclusion)* |  |  |  |
| (15) | Is there any extensive use of emotive language by the CDE? *(feel, sad, happy, depressed)* |  |  |  |
| (16) | Do CDEs or patients use certain words that may cause offence? *(fat instead of overweight, is it cruel/on purpose, racist, homophobic, avoiding words- how does CDE cope with it?)* |  |  |  |
| (17) | Assess the body language of the CDE? *(positive- hand gestures, negative- arms crossed, does it change through the session)* |  |  |  |
| (18) | How clearly does the CDE communicate? *(are they audible, clear, loud, draws attention to themselves)* |  |  |  |
| (19) | Overall what are the CDEs strong qualities and what do they find challenging/difficult? |  |  |  |
| Structure of sessions/ programme  (20) | Are the aims and objectives clearly stated at the beginning of the session?  How is this done? |  |  |  |
| (21) | What is the teaching style adopted by the CDE? *(didactic, participative)* |  |  |  |
| (22) | Does the teaching style change throughout the session? *(for diff. groups, speed things up, due to pt. needs-clarity)* |  |  |  |
| (23) | Do teaching styles differ between CDEs that are bilingual and non-bilingual? *(how- more hand movements, pt involvement, greater structure, ‘loose’ format)* |  |  |  |
| (24) | Are the sessions predominantly CDE led or group led in the session? |  |  |  |
| (25) | Is there significant time spent in recording patient data? *(allocation of time to info, education, recording, activities)* |  |  |  |
| Theoretical Questions (notes for debriefing)  (26) | Is the programme a good application of the HBM, SCT, TPB, SDT or TTM? *(outline of the costs and benefits by pt)* |  |  |  |
| (27) | Self-Efficacy: is there clear identification of outlining personal goals, patient evaluation, how high/low is patient confidence? *(inclusion of any social factors)* |  |  |  |
| (28) | Are power relations clearly identifiable within the session? |  |  |  |
| (29) | Do sessions outline the role of cultural norms and practices? How is this done? |  |  |  |
| (30) | Issues/Factors relevant to the ‘Patient Experience’ of the CDE programme. |  |  |  |
| (31) | Additional Information |  |  |  |
