# Supplementary material for: A critique of the design, implementation, and delivery of a culturally-tailored self-management education intervention: a qualitative evaluation
Source: BMC Health Serv Res. 2015 Feb 7;15:54. doi: 10.1186/s12913-015-0712-8 (PMC4326406; doi:10.1186/s12913-015-0712-8)
Supplement: Additional file 4: — Themes and supporting quotes. [file 12913_2015_712_MOESM4_ESM.doc]

**Additional file** 4: Themes and supporting quotes

| **Themes** | **Supporting quotes** |
| --- | --- |
| **Findings: Lay educators** | they just ask me, “is this true, is this true”, I go this is what’s true and some are myths, so try not to follow the myths just the facts (CDE, interview).  Their [White British and Black/Black Caribbean] mentality is slightly different to the Asian culture so you know they don’t eat many spices anyway, their lifestyles, their little habits. I find that’s its easier for them to make the changes than it is of Asian people because they just have their set plans you know, you make one dish and you have it twice a day, and everybody has the same and you have your chapattis (CDE, interview).  We show them the traffic light system, like types of food, so instead of using English brands I use Asian brands, like East End (CDE, interview).  if it was Muslim women, sometimes they prefer a women only group so you have to be sensitive to that (CDE, interview).  the visual aids aren’t culturally relevant, like with the food plate the oils and fats that we’ve got, even the bits of salt aren’t culturally relevant… the oils we purchased from an outside agency, the salt’s again have been purchased by an outside agency as well, so it’s what they provide (CDE, interview).  the interpreter that I work [with], they (South Asian community) really value her, they do, I suppose they’re also seen as part of her community as well. Us working alongside together has been, very positive. I suppose it has broken down perhaps a barrier that may have been between me and the group in a way, if that was ever felt (CDE, interview).  I have to be very strict and say I’m going to do a language only course, cause it’s very hard to chop and change, we tried it and it doesn’t work…cause it would be a lot easier delivering it in a community language than it is delivering half in a community language then in English and back into a community language, cause people just get frustrated, they get tired (CDE, interview).  It’s quite tiring because working with Asian people is a challenge, because I find it very challenging, because it’s a very tough community to work with and especially trying to send out the message that we want, because they’re so set in their ways (CDE, interview).  they’ve all got their individual problems, for example Jamaican’s they tend to use a lot of salt on their salt fish and everything, you’ve got Asian’s with the fat and the *ghee* [clarified butter] (CDE, interview). |
| **Findings: Observations for fidelity** | The use of the visual aids (images of food portions and the sugar bags- identifying how much sugar is in different types of food) makes a considerable impact on providing a complete picture and makes the content very relevant. PT (1) responds in English, “My God”- PT (5) responds in Punjabi and English combined when she sees a picture of some ice cream, “*bhoort tasty*” [*very tasty*]- CDE picks up image and replies that it is very sugary and unhealthy- PT (1) takes sugar bag- PT (3) in Urdu, “*herani*” [shocked/amazed] (CDE, observational notes).  No recap of the previous week- all participants present in this session were present in the previous session- PT 3,6,8,9,13 bring their food dairies to the session- CDE does have a look at these diaries, however, gives the diaries back to them, “it’s for you guys to use really”. Some appear to think that it was bit of a useless task, maybe wished to gain more feedback from the CDE; advice on where changes could be made, more knowledge and guidance (CDE, observational notes).  CDE goes round the table- not asking everyone- on what changes they have made since they began the course- PT 3- “look at labels, more oats, trying to cut down the salt” PT 2-“I’ve learnt a lot, cut down on the chocolate, started telling everyone else” (CDE, observational notes).  Discussion about tension [stress], thoughts and sadness. CDE informing PT 1 to take care of his blood pressure- caring daughter approach (CDE, observational notes).  PTs are very interactive with those who are closest to them (PT 13, 7, 6) (PT 8 and 9) and (PT 2 and 3): small groups have formed. PT 6,7 very interactive- PT 5 not present- PT 13 joins this group- females of her age- feels she can relate to them better. PT 13 asks PT 7 if she would like to go to the gym with her- has no one to go with- PT 7 declines- problem with self-image- in front of others (CDE, observational notes).  PT 2 and 3 (both male)- in conversation for a long time- conversations lead to questions directed to the CDE- all related to the discussion- salt causing high BP- giving each other more information (CDE, observational notes).  CDE stated that going out, socialising, and going to church encourages positive well-being. Participants already confident of undertaking tasks that will improve their emotional well-being (CDE, observational notes)  The use of persuasion. Outlining the benefits of undertaking certain tasks such as exercise which can improve stress management and (CDE) stating to the participant more exercise will result in a positive outlook; participant nods in agreement (CDE, observational notes).  Sorry, so what’s being said? (CDE, observational notes).  PT 5 states she did not understand the translation delivered to her by the interpreter- firstly in Punjabi, and then does it in Urdu- slightly better. PT 5 speaks Bengali- not spoken by the interpreter which did make her slightly isolated (CDE, observational notes). |
| **Findings: The participant experience** | The impression I got with him … I was a bit apprehensive about him, but when he started talking … I think if you need to ask him, he was there to ask (CDE-ATT-17, Male, White British, T2D and heart conditions).  [Interviewer] So, was it of any benefit that [CDE] was from a similar background?  [Respondent] No, whoever’s a good person, like we speak to white people, we speak to Black people, so what’s wrong if everybody is friendly with each other (CDE-ATT-13, Female, Asian Indian, heart conditions).  she was telling everyone in Punjabi, do this, do that, she spoke good Punjabi, sometimes she spoke in English, everything was fine with her (CDE-ATT-11, Female, Asian Indian, T2D and Hypertension).  He was mostly happy with the fact that on how she[CDE] advised on how to lose the weight, how to minimise your food [consumption] and stuff because he wasn’t very aware of it (CDE-ATT-07, Male, Asian Bangladeshi, T2D, via interpreter).  No, no it didn’t feel like a classroom at all, no, it was just like you were going in to have a talk with a normal friend in a friendly atmosphere (CDE-ATT-03, Female, White British, T2D).  if you ask another person with diabetes, he will tell you his experience, you could ask a hundred people, if you talk to a hundred people they all give you their advice, of their experience, but sometimes it doesn’t work with you, you know (CDE-ATT-15, Male, Asian Indian, T2D).  Because we’re all individuals, what suits me, they could not copy me because it may not suit them (CDE-ATT-04, Female, Black Caribbean, T2D).  The whole condition was brought out in the group because people were at different stages with their diabetes and on different medication and they were experiencing different things because a lot of them, or some of them, had different health issues, medical issues, as well as their diabetes (CDE-ATT-10, Female, Black Carribbean, T2D). |
